# Classification of Motor Imagery EEG Signals with Support Vector Machines and Particle Swarm Optimization

**DOI:** 10.1155/2016/4941235

**Published:** 2016-05-30

**Authors:** Yuliang Ma, Xiaohui Ding, Qingshan She, Zhizeng Luo, Thomas Potter, Yingchun Zhang

**Affiliations:** ^1^Institute of Intelligent Control and Robotics, Hangzhou Dianzi University, Hangzhou, Zhejiang 310018, China; ^2^Department of Biomedical Engineering, University of Houston, Houston, TX 77204, USA; ^3^Guangdong Provincial Work Injury Rehabilitation Center, Guangzhou 510000, Chinas

## Abstract

Support vector machines are powerful tools used to solve the small sample and nonlinear classification problems, but their ultimate classification performance depends heavily upon the selection of appropriate kernel and penalty parameters. In this study, we propose using a particle swarm optimization algorithm to optimize the selection of both the kernel and penalty parameters in order to improve the classification performance of support vector machines. The performance of the optimized classifier was evaluated with motor imagery EEG signals in terms of both classification and prediction. Results show that the optimized classifier can significantly improve the classification accuracy of motor imagery EEG signals.

## 1. Introduction

Brain-computer interface (BCI) is a new technology based on electroencephalographic (EEG) signals [1] that provides a new way for the patients with motor dysfunction to communicate with the outside world. In a BCI system, electrical activity within the cerebral cortex is detected through the use of an electrode cap and other equipment, and the motor imagery EEG signals are converted into instructions to control an external device [2]. In recent years, BCI has been a major focus in the field of brain science and biomedical engineering and has been developed into a new multidisciplinary cross technology.

The key component of a BCI system is how it extracts EEG characteristics to improve the recognition accuracy, as the accuracy of this pattern recognition directly affects the performance of the system. At present, the most commonly used pattern recognition methods include the Linear Discriminant Analysis (LDA), *k*-Nearest Neighbor (KNN) classification algorithm, Artificial Neural Network (ANN), and Naïve Bayes (NB). The support vector machine (SVM) was first developed in 1995 by Vapnik based on statistical learning theory, which is usually used for classification and nonlinear regression. The main idea of SVMs is to change the vector to a higher dimensional space and obtain the optimal classification plane by calculation so as to make the sample linearly separable. Kor Shoker has previously analyzed the corresponding characteristic parameters of the event-related synchronization (ERS) and event-related desynchronization (ERD), using the SVM classifier for classification. Because SVMs can solve the practical problems associated with small sample sizes, nonlinear relationships, high dimensions, and local minima the machines can achieve classification accuracy above 83.5%. However, the kernel function of SVM is often difficult to choose in practical applications due to the random and nonstationary nature of EEG signals and the lack of prior knowledge regarding the distribution characteristic of the signal [3].

In traditional SVM classifiers, the penalty and nuclear parameters are usually chosen according to the empirical data, ignoring the importance of optimizing the parameter to enhance the effects of the classifier. To improve this, a particle swarm optimization (PSO) algorithm has been applied to select the best kernel function and penalty parameters and thereby improve the accuracy of classification [4, 5]. The combination of PSO and SVM was used by Maali and Al-Jumaily for the recognition of apnoea and other sleep dysfunctions [4] and by Subasi for optimizing the SVM for EMG signal classification [5].

The classification accuracy of SVM largely depends on the selection of the kernel function parameters. By combining with the characteristic of EEG signals, the PSO can be used to optimize the parameters of the kernel function and the penalty parameter of EEG SVM classification and thereby improve classification accuracy.

## 2. Feature Extraction

### 2.1. Experiment Data 1

The experimental motor imagery EEG data was obtained from the 2005 competition, BCI Competition III Data Iva, which has been published by the German Research Center for BCI movement. Databases from five healthy subjects (aa, al, av, aw, and ay aged 24 to 25 years) were obtained, in which the subjects conducted left hand, right hand, or right foot motor imagery. The right hand and right foot imagery data was isolated for analysis within this paper.

**Figure 1:**
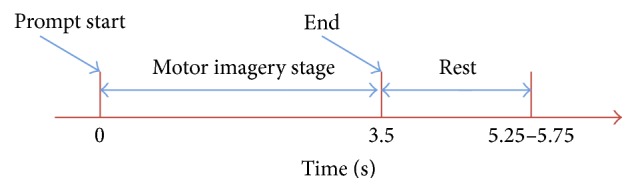
The timing chart of a single motor imagery experiment.

The specific collection process of the experiment was as follows: the five subjects were asked to sit quietly on the chair and perform the corresponding imagery according to prompts shown on a screen in front of them. The timing diagram of a single imagery experiment can be seen in Figure 1. Once prompts were given, the subject performed the appropriate motor imagery for 3.5 seconds and rested afterwards for a variable time of 1.75–2.25 seconds. During collection the electrodes were placed in the standard 10–20 lead mapping, using 59 channels recorded at a sampling frequency of 100 Hz. Every subject conducted four sets of experiments, each containing 70 runs of motor imagery data for a total of 280 runs per subject. The specific data of the five subjects is shown as in Table 1.

### 2.2. Experiment Data 2

The experimental motor imagery EEG data was obtained from the 2005 competition, BCI Competition III Data Iva, published by the German Research Center for BCI movement. The data comes from seven healthy subjects, alphabetically labeled a–g. During the experiment the subjects completed motor imagery of left hand, right hand, or foot according to the computer prompts. Subjects a and f were asked to perform left hand and foot imagery while the remaining five subjects performed imagery of the left and right hands. EEG electrodes were placed in the standard 10–20 lead mapping and data collection featured 59 channels with a sampling frequency of 100 Hz. The experimental data from subjects a, b, f, and e have been analyzed in this paper.

**Figure 2:**
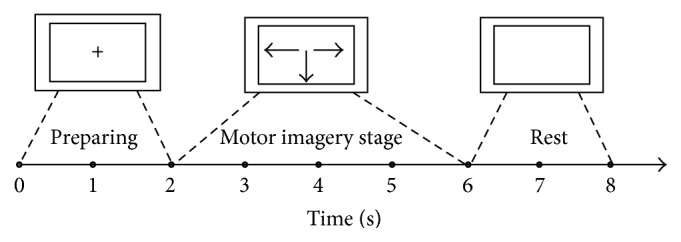
The timing chart of a single motor imagery experiment.

In the experiment subjects performed 200 motor imagery tasks, each lasting for 8 s. During the task, a cross was first presented for 2 s, indicating that the subject should prepare for motor imagery. Subjects were then shown an arrow pointing left, right, or down for 4 seconds, indicating that the subject should imagine respective motion of the left hand, right hand, or foot. Finally, subjects rested for 2 seconds while a blank screen was shown. The full experimental process can be seen in Figure 2.

### 2.3. Common Spatial Pattern

Common spatial pattern (CSP) is an improved algorithm based on principle component analysis. While it has been mainly used in face recognition, object recognition, and EEG anomaly detection, it has also been successfully applied to brain-computer interfaces. The CSP algorithm is a kind of multidimensional statistics that has often been applied to EEG signal feature extraction and analysis in two-class multichannel methods. According to the phenomena of ERD/ERS during motor imagery, feature extraction using CSP is as follows: firstly, the two classes of motor imagery EEG signal will be filtered by CSP to make a class of signals (such as left hand motor imagery) with maximum variance and a class (such as right hand motor imagery) that features minimum variance, making a clear distinction between the two groups. The following are the specific steps of EEG signal feature extraction using CSP.

Suppose that the motor imagery EEG signal is expressed as *E*_*j*_^*i*^, where *i* is the sample and *j* represents one of the two different categories with the range *j* ∈ {1,2}. The dimension of the EEG signal is *N* × *T*, where *N* is the number of the channels and *T* is the number of samples recorded in the experiment. The steps for creating the two classes of motor imagery for EEG signal feature extraction are then as follows.

(1) Calculate the sample covariance of the two classes of motor imagery:

(1) $$\begin{array}{c} R_{j}=\frac{E_{j}^{i}E_{j}^{iT}}{trace\left(E_{j}^{i}E_{j}^{iT}\right)}, \end{array}$$

where *E*_*j*_^*iT*^ is the transpose of *E*_*j*_^*i*^, trace(*E*_*j*_^*i*^ *E*_*j*_^*iT*^) is the trace of the matrix, and *R*_*j*_ represents the covariance of class *j*. The space covariance can be calculated as the average of all the same class data covariance of EEG. Supposing that the average covariance values of the two-class motor imagery sample are indicated as $\bar{R_{1}}$ and $\bar{R_{2}}$, then the general covariance can be expressed as

(2) $$\begin{array}{c} R_{c}=\bar{R_{1}}+\bar{R_{2}}. \end{array}$$

(2) Feature decomposition of *R*_*c*_ by principle component analysis is as follows:

(3) $$\begin{array}{c} R_{c}=B\lambda B^{T}, \end{array}$$

where *B* is the eigenvector matrix and *λ* is the corresponding characteristic values. The variance can be then uniformed by a whitening matrix, and the whitening matrix *P* is defined as

(4) $$\begin{array}{c} P=\lambda^{-1/2}B^{T}. \end{array}$$

The average covariance of the two classes of motor imagery EEG signal can then be changed as follows with the whitening matrix *P*:

(5) S1=PR1¯PT,S2=PR2¯PT.

(3) The covariance matrix of the two classes has the same eigenvectors:

(6) S1=Uλ1UT,S2=Uλ2UT.

So the corresponding feature values of the two classes of covariance matrices can be obtained, which have been expressed here as the values *λ*_1_ and *λ*_2_ that satisfy the equation *λ*_1_ + *λ*_2_ = *I*, where *I* is an identity matrix. Therefore, according to the above formula, if the feature value of *S*_1_ is maximal, then the feature value of the corresponding feature vector of *S*_2_ is minimal, and vice versa, two classes of the EEG signal are separable.

(4) According to step (3), *U* can be used to distinguish the two different kinds of dataset. The first-*m* and last-*m* representative feature vectors can be chosen to form a matrix from the two types of the feature vectors and the projection matrix can be expressed as

(7) $$\begin{array}{c} W=B^{T}P. \end{array}$$

Then the projected matrix of the two types of the motor imagery EEG signal through the filter can be expressed as

(8) $$\begin{array}{c} Y=WE_{j}^{i}. \end{array}$$

Finally, the feature of the coefficient of the logarithm can be indicated as

(9) $$\begin{array}{c} f_{j}=\log\left(\;\frac{var\left(Y_{j}\right)}{\sum_{k=1}^{k=2m}\log\left(var\left(Y_{k}\right)\right)}\;\right),\;j=1,2,\ldots ,2m, \end{array}$$

where *Y*_*j*_ is the *j*th line of *Y* and var(*Y*_*j*_) is the variance.

### 2.4. Regularized Common Spatial Pattern

In traditional CSP EEG feature extraction, the data is recorded from only one subject. When the sample size for this subject is small, the feature extraction results are often unsatisfactory, especially considering how easily EEG data can be influenced by the emotions or physical conditions of the subjects. In order to solve this problem, the Regularized Common Spatial Pattern (RCSP) algorithm has been used for feature extraction [6, 7]. This method introduces a regularization parameter to avoid the drawbacks of a single sample and reduce individual differences. The specific procedures are as follows.

*n* subjects are selected, specifying one as the main subject and the others as secondary subjects. The regulation parameters of *β* and *γ*  (0 ≤ *γ*, *β* ≤ 1) are then introduced to combine the covariance matrix of the main subject with those of secondary subjects, and the two classes of the covariance matrix are constructed as follows:

(10) Z1γ,β=1−γ1−β·R1+β·R11−β·m+β·n−1·m+γNtr1−β·R1+β·R11−β·m+β·n−1·m·I,Z2γ,β=1−γ1−β·R2+β·R21−β·m+β·n−1·m+γNtr1−β·R2+β·R21−β·m+β·n−1·m·I,

where *R*_1_ and *R*_2_ represent the two-class sample covariance matrices of the main subject, the covariance matrices of the other *n* − 1 subjects are *R*_1_ and *R*_2_, *m* is the number of the experiments, and tr express the trace of the matrix. *I* is a unit matrix of *N* × *N*, where *N* is the number of the channels. The two-class covariance matrices are solved and the feature decomposition formula performed is as follows:

(11) $$\begin{array}{c} Z\left(\;\gamma ,\beta \;\right)=Z_{1}\left(\;\gamma ,\beta \;\right)+Z_{2}\left(\;\gamma ,\beta \;\right)=U\Lambda U^{T}, \end{array}$$

wherein *U* is the feature vector, Λ is the diagonal matrix of the corresponding eigenvalues, and the whitening matrix is defined as

(12) $$\begin{array}{c} P={\hat{\Lambda }}^{-1/2}\cdot \hat{U^{T}}. \end{array}$$

Equation (10) is subsequently whitened:

(13) Z¯1γ,β=P·Z1λ,β·PT=U1·Λ1·U1T,Z¯2γ,β=P·Z2λ,β·PT=U2·Λ2·U2T,

wherein Λ_1_ and Λ_2_ are the eigenvalues and *U*_1_ and *U*_2_ are the feature vectors. Selecting the first- and last-*m* eigenvectors of the eigenvalues, then the spatial filter is constructed as follows:

(14) W1=U1T·P,W2=U2T·P.

The training samples of *X*_1_ and *X*_2_ are extracted by CSP and the feature vector of [*Z*_1_, *Z*_2_] is obtained as follows:

(15) Z1=W1X1,Z2=W2X2.

## 3. Feature Recognition of EEG

### 3.1. Support Vector Machine

Support vector machines were created by Vapnik based on the statistical learning theory and can solve the problems associated with small sample sizes, nonlinear relationships, and multiple classifications [8]. The principle of SVM classification is to construct an optimal hyperplane as the decision surface to identify the different classes, so as to maximize the spacing between them.

In solving the nonlinear classification problem of SVM, the kernel function is used instead of the inner product computation and nonlinear problems are converted to linear classification problems by raising their dimension [9]. The paper mainly focuses on two-class feature classification, and the sample set is expressed as (*x*_*i*_, *y*_*i*_), *i* = 1,2,…, *l*, *x* ∈ *R* ^*N*^, where *y*_*i*_ ∈ {−1, +1} is the identifier of the category. The discriminant function can be expressed as

(16) $$\begin{array}{c} y_{i}\left[\;\left(\;w\cdot x_{i}\;\right)+b\;\right]-1\geq 0,\;i=1,2,\ldots ,l. \end{array}$$

Through the Lagrange multiplier, this can be transformed into a dual problem and the conversion optimization objective function is as follows:

(17) min⁡ Qa=12∑i,j=1laiajyiyj·Kxi,xj−∑i=1lai,

which satisfies the constraints: ∑_*i*=1_^*l*^ *a*_*i*_ *y*_*i*_ = 0, 0 ≤ *a*_*i*_ ≤ *C*, where *a*_*i*_ represents the corresponding Lagrange multipliers of *M* for each constraint and *C* is the punishment parameter of the sample. To solve the above problem, an appropriate kernel function *K*(*x*, *y*) is selected and a RBF function is used chosen in this paper, which can be expressed as

(18) $$\begin{array}{c} K\left(\;x,x_{i}\;\right)=\frac{\exp\left(-{\left|x-x_{i}\right|}^{2}\right)}{g^{2}}. \end{array}$$

The optimal classification function can be obtained as

(19) $$\begin{array}{c} f\left(\;x\;\right)=\mathrm{sgn}\left(\;\overset{l}{\underset{i=1}{\sum }}a_{i}^{*}y_{i}K\left(\;x,x_{i}\;\right)+b^{*}\;\right), \end{array}$$

wherein *a* ^*∗*^ and *b* ^*∗*^ are the parameters used to determine the optimal classification surface, which can be obtained by a support vector.

### 3.2. Particle Swarm Optimization

The particle swarm optimization (PSO) approach was proposed by Kennedy and Eberhart in 1995 as an evolutionary optimization algorithm [10, 11]. The main principle of PSO is to start with a random point and then evaluate the fitness function of that particle which will be evaluated to find the optimal solution by iterative optimization [12]. The primary characteristics of PSO are sample structure, a relatively low number of adjustment parameters rapid convergences, and ease of implementation, leading to the broad use of the algorithm in various fields.

PSO is a global iterative optimization algorithm. Each individual in the population is represented as a particle. At the beginning a fitness function is set to determine the fitness value of each particle and these particles are set to move within the search space according to their speed and position following the current of optimal moving particles [13]. Following this process, a final optimal solution will be obtained. During each iteration the particle will move along a track that is optimal both for itself and for the group. The specific optimization process is described as follows.

Supposing in dimensional space *d* there are *i* particles, the position (*x*_*i*_) and velocity (*v*_*i*_) of each particle are *x*_*i*_ = (*x*_*i*1_, *x*_*i*2_,…, *x*_*id*_)^*T*^ and *v*_*i*_ = (*v*_*i*1_, *v*_*i*2_,…, *v*_*id*_)^*T*^, where *i* = 1,2,…, *N*. The best position of each particle *i* is *p*_*i*_ = (*p*_*i*1_, *p*_*i*2_,…, *p*_*id*_), and the extreme values of the global populations are *p*_*g*_ = (*p*_*g*1_, *p*_*g*2_,…, *p*_*gd*_). Each particle then adjusts their own speed and position by comparing with the global and individual extreme value to get the optimal parameters through iterative calculation. The particle update formulae for velocity and position are expressed as follows:

(20) vidt+1=vidt+c1r1pidt−xidt+c2r2pgdt−xgdt,

(21) xidt+1=xidt+vidt+1,

wherein the position *x*_*id*_^*t*^ ∈ [*L*_*d*_, *U*_*d*_] and *L*_*d*_ and *U*_*d*_, respectively, represent the lower and upper *D*-dimensional space. The current iteration number is given by *t* and the range of the speed is *v*_*id*_^*t*^ ∈ [*v*_min,*d*_, *v*_max,*d*_], where *v*_min,*d*_ and *v*_max,*d*_ are the lower and upper particle velocity. *r*_1_ and *r*_2_ are random numbers uniformly distributed in (0,1) and *c*_1_ and *c*_2_ are the constants called learning factors, which trade off the best position for the particle itself and the best position among groups. Shi and Eberhart added the inertia weight parameters to the original PSO to control the search scope and to reduce the importance of the upper speed limit. Using this, formula (20) can be changed to

(22) $$\begin{array}{c} v_{id}^{t+1}=wv_{id}^{t}+c_{1}r_{1}\left(\;p_{id}^{t}-x_{id}^{t}\;\right)+c_{2}r_{2}\left(\;p_{gd}^{t}-x_{id}^{t}\;\right), \end{array}$$

where the greater the value of the inertia weight parameter *w* is, the stronger the capability of the global search becomes. In contrast, the local search ability becomes weaker. Lastly, *t* is the number of the iterations and once the termination condition is reached, the optimal solution will be obtained.

### 3.3. Improved PSO-SVM Classifier

The selection of the kernel parameter for the support vector machine can directly affect the performance of the classifier, making this selection process quite important. The traditional cross-validation method for selecting this parameter, however, is inefficient and will not necessarily achieve an optimal solution. Due to the parallelism and the independence in the target optimization of PSO, the method can be successful in solving the nonlinear problems in high-dimensional space [14].

**Figure 3:**
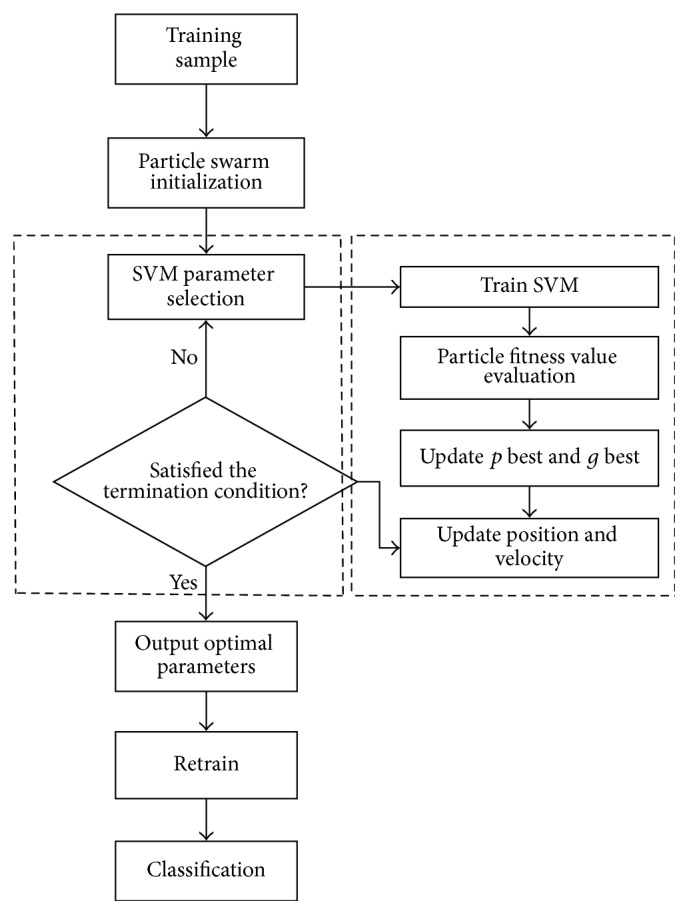
The flowchart of PSO optimized SVM parameters.

The RBF function has been chosen here as the kernel function of SVM for the pattern classification of motor imagery EEG signals. SVM classification relies heavily upon the selection of appropriate penalty and kernel parameters. These parameters will be optimized by PSO, and the optimal parameters will be applied to EEG signal classification and prediction. The flowchart of the optimized SVM based on the PSO algorithm is shown in Figure 3, and the concrete steps are as follows.

*(1) Initialization*. In the *D*-dimensional parameter space, the position and the velocity of the particle *M* will be initialized, including setting the initial parameters of *c*_1_ and *c*_2_, as well as the inertia weight of the population. The penalty parameter and the nuclear parameters of the SVM will be initialized, and the size of the population and the largest number of the iterations will be determined.

*(2) Calculating the Fitness*. First model the support vector machine with the initialized parameters, and train the model based on the training sample. Then calculate the fitness function values using the fitness function of each particle.

*(3) Adjusting*. Adjust the personal best position and the global best position according to the particle fitness value.

*(4) Updating*. Update the position and the velocity of the particle according to formulae (21) and (22) to get the new parameters of *p* best and *g* best.

*(5) Determination*. When the error condition or the maximum number of iterations has been reached, stop the iterative output. Otherwise, return to step (3) to continue the calculation.

*(6) Classification*. Output the optimal kernel parameter *g* and the penalty factor *C*; then, retrain the SVM classifier with the training sample. Finally, use the obtained best classifier for class prediction.

Through the six steps above, complete the parameter optimization of the SVM penalty parameter and kernel parameter based on PSO, and use the optimized classifier for the classification and prediction of motor imagery EEG signals.

## 4. Experimental Results and Analysis

### 4.1. The Process of PSO Optimizing the SVM Parameters

In this experiment, the data obtained from BCI Competition 2005 Data Iva has been applied for analysis. The five subjects of aa, al, av, aw, and ay have been chosen for the feature extraction and classification experiment, and the C3 and C4 channels have been selected for the analysis of motor imagery EEG data.

**Figure 4:**
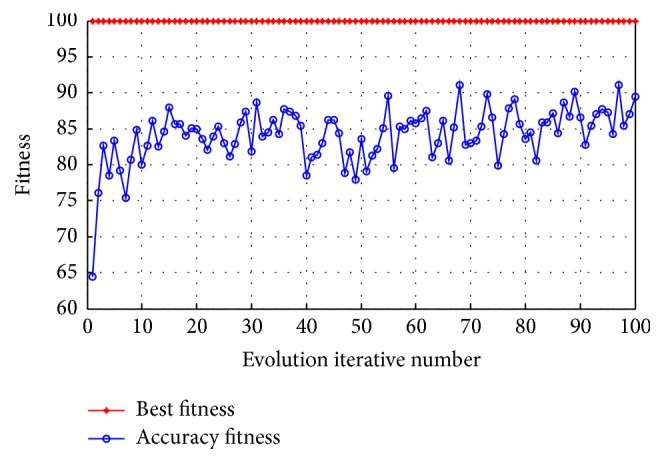
The fitness curve of the particle swarm optimization parameters.

Firstly, the CSP method was applied for feature extraction for the two classes of the motor imagery EEG signal. Then, the PSO-SVM classifier was used to classify the extracted feature vectors. The initial parameters of the PSO were set as *w* = 0.8, *c*_1_ = 1.5, and *c*_2_ = 1.7. The 50 particles were used in the swarm, the maximum number of iterations was set to 100, the penalty parameter *C* was in the range of (0.1, 100), and the kernel parameter *g* was in the range of (0.01, 1000). The experimental iterative optimization process for subject aa is shown in Figure 4. As shown in the figure, the continuous iteration process will gradually approach maximal fitness. When the termination condition was reached, the average fitness was approximately 90% and the output values of the optimal parameters were *C* = 4.5294 and *g* = 0.01.

### 4.2. Experiment 1 Classification Results and Analysis

**Figure 5:**
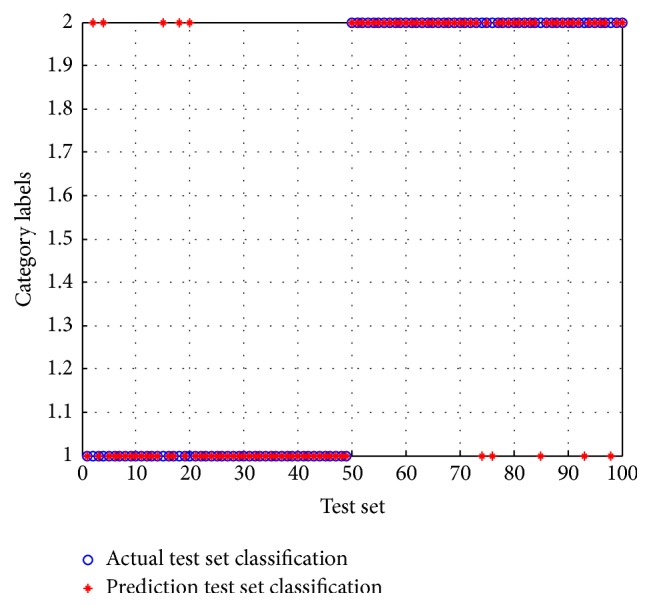
The classification accuracy figure before optimization.

**Figure 6:**
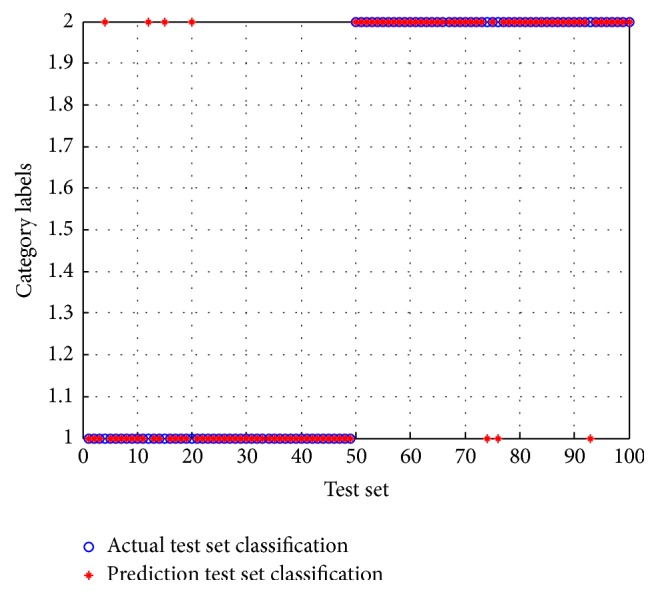
The classification accuracy figure after optimization.

In the experiment of the BCI Competition 2005 Data Iva, one subject was selected as the main subject while the four remaining subjects were selected as the supplementary subject, with the number of samples for each selected as 200, 100, 80, and 56 in decreasing order. The ratio training samples to test samples were 60 : 40. In order to achieve the best feature classification results, the appropriate regulation parameters needed to be selected. After several tests, the regularization parameters were set to zero for subjects aa and al, while the parameters were set to *β* = 0 and *γ* = 0.01 for subjects av and aw to reach the ideal feature extraction result. Figures 5 and 6, respectively, represent the classification rate of subject aa with the SVM and PSO-SVM classifiers. When the test label coincides with the pretest label, it indicates that the classification results are the same in that type of imagined motion. In these figures, it is clear that the classification accuracy after PSO is greater than that obtained without optimization, indicating an overall improvement in performance.

To verify that the recognition results of the SVM after the PSO were optimized, subjects al, aa, av, and aw were selected for motor imagery EEG signal classification with both the traditional SVM and PSO-SVM classifiers. The classification accuracy results of the four subjects with SVM and PSO-SVM are shown in Table 2. Each of the four subjects showed increases in both maximum and average classification accuracy. The average recognition of the four subjects increased about 2%, indicating that the PSO-SVM can effectively improve the performance of the SVM classifier when optimal parameters have been obtained.

**Figure 7:**
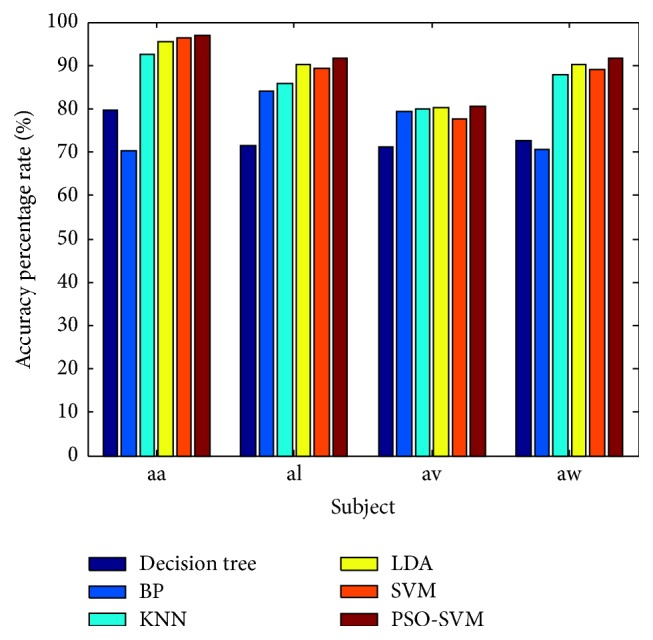
The classification accuracy of PSO-SVM compared with traditional methods for 2005 Data Iva.

To further verify the validity of the PSO optimized SVM, the classification results of PSO-SVM have been compared with the traditional classification methods such as decision tree, BP, KNN, and LDA. The classification results of LDA are from the literature [12]; subjects aa, al, av, and aw were chosen as the main subjects with the remaining four chosen as the secondary subjects. In this experiment, the first 40 sets of experimental data were chosen from each subject and the regularization parameters were set as *β* = 0 and *γ* = 0.01. The maximum and average classification accuracy results of the methods are shown in Table 2. In order to compare the classification performance of different classifiers clearly, the average classification accuracy results are shown in Figure 7. This bar chart, comparing the classification result of PSO-SVM with traditional classification methods, shows that the classification accuracy of PSO-SVM is higher than that of other methods in each subject, with classification accuracy reaching up to 97%. The final results then show that the PSO-SVM has clear advantages and it can effectively improve EEG classification accuracy.

### 4.3. Experiment 2 Classification Results and Analysis

In this experiment, the data of BCI Competition Datasets 1 2008 have been applied for analysis. Four subjects, a, b, f, and e, have been chosen for the feature extraction and classification experiment and the C3 and C4 channels selected for the analysis of motor imagery EEG data. The data of the four subjects were firstly extracted feature by the CSP method. After 100 experimental iterations, the final classification results of the PSO-SVM and SVM were obtained. The results from these classifications are shown in Table 3.

From this table, the accuracy results again show that the PSO optimized SVM classifier can improve the classification rate for two-class EEG signal in most cases. While subject a was able to achieve a marginally greater maximum classification accuracy with standard SVM, all other subjects and conditions show increased performance with the PSO-SVM algorithm. The CSP method combined with the PSO-SVM classifier can subsequently be considered more suitable for motor imagery EEG classification.

**Figure 8:**
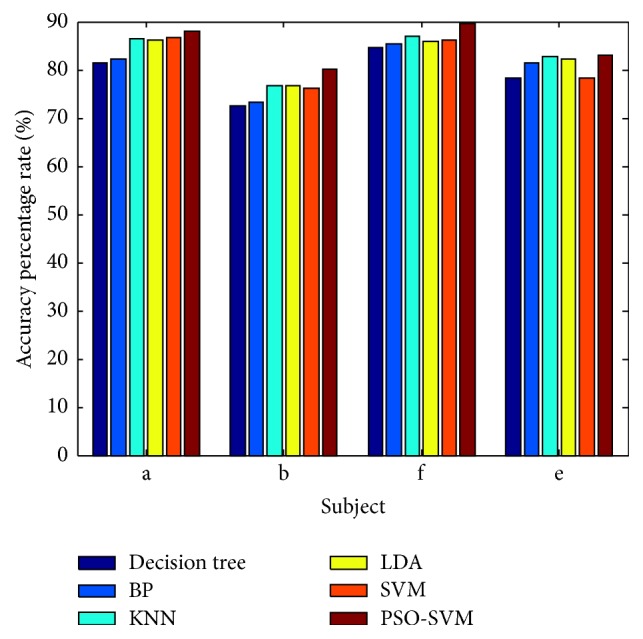
The classification accuracy of PSO-SVM compared with traditional methods for 2008 Dataset 1.

Furthermore, in order to verify the validity of the PSO optimized SVM, the classification results of PSO-SVM have been compared with the traditional classifiers such as decision tree, BP, KNN, and LDA. In this experiment, four subjects a, b, f, and e were firstly extracted feature by the CSP method. The maximum and average classification accuracy results of the methods are shown in Table 3. In order to compare the classification performance of different classification methods clearly, the average classification accuracy results are shown in Figure 8. This bar chart, comparing the classification result of PSO-SVM with traditional classifiers, shows that the classification accuracy of PSO-SVM is higher than that of other methods in each subject, with classification accuracy reaching up to 89.7%. The final results show that the PSO-SVM algorithm has clear advantages for EEG classification.

## 5. Conclusions

This paper has focused on the classification of the motor imagery EEG signals by analyzing the data obtained from the BCI Competition Datasets 1 2008 and 2005 competition BCI Competition III Data Iva. The final experimental results show that the kernel function of SVM optimized by particle swarm optimization can effectively improve the classification accuracy. The PSO-SVM classification method is able to overcome the shortcomings of the parameter selection problem that traditional SVM is subject to. The systematic nature of PSO-SVM further allows for the rapid determination of parameters, making the process much less time-intensive. In a word, the PSO-SVM classifier can reach a better accuracy rate than traditional classification method.

## Figures and Tables

**Table 1:** The experiments number of the five subjects.

Subjects	Training times	Test times
aa	168	112
al	224	56
av	84	196
aw	56	224
ay	28	252

**Table 2:** The classification results of PSO-SVM and traditional methods for 2005 Data Iva.

Accuracy (%)	aa (100)		al (200)		av (80)		aw (56)	
	Max	Average	Max	Average	Max	Average	Max	Average
Decision tree	82.5	79.8	74.6	71.5	74.2	71.2	75.4	72.8
BP	71.4	70.2	85.8	84.2	81.6	79.4	72.2	70.6
KNN	94.6	92.5	87.6	85.8	82.4	80.1	89.2	87.8
LDA	96.2	95.4	92.7	90.2	82.8	80.2	91.6	90.2
SVM	97.7	96.4	92.5	89.4	81.5	77.6	90.5	88.9
PSO-SVM	98.1	97.0	93.9	91.7	82.0	80.5	92.3	91.6

**Table 3:** The classification results of PSO-SVM and traditional methods for 2008 Dataset 1.

Accuracy (%)	a		b		f		e	
	Max	Average	Max	Average	Max	Average	Max	Average
Decision tree	84.5	81.4	74.6	72.5	86.2	84.5	84.6	78.2
BP	85.6	82.2	74.8	73.2	87.6	85.4	83.4	81.5
KNN	90.6	86.5	78.6	76.8	91.4	87.1	84.2	82.8
LDA	89.4	86.2	80.5	76.6	90.8	86.0	86.4	82.2
SVM	91.7	86.8	78.0	76.3	95.0	86.1	90.5	78.3
PSO-SVM	91.3	88.1	83.5	80.1	95.2	89.7	92.0	83.1
